# Sex differences in health burdens across the lifespan in wild chimpanzees (*Pan troglodytes schweinfurthii*)

**DOI:** 10.1093/emph/eoaf031

**Published:** 2025-11-05

**Authors:** Elizabeth V Lonsdorf, Margaret A Stanton, Ian C Gilby, Thomas R Gillespie, Zarin P Machanda, Deus Mjungu, Martin N Muller, Dismas Mwacha, Emily Otali, Richard W Wrangham, Melissa Emery Thompson

**Affiliations:** Department of Anthropology, Emory University, Atlanta, GA, USA; School of Social and Behavioral Sciences, University of New England, Biddeford, ME, USA; School of Human Evolution and Social Change, and Institute of Human Origins, Arizona State University, Tempe, AZ, USA; Department of Anthropology, Emory University, Atlanta, GA, USA; Department of Environmental Sciences, Emory University, Atlanta, GA, USA; Department of Environmental Health, Rollins School of Public Health, Emory University, Atlanta, GA, USA; Department of Anthropology, Tufts University, Medford, MA, USA; Gombe Stream Research Centre, The Jane Goodall Institute, Kigoma, Tanzania; Department of Anthropology, University of New Mexico, Albuquerque, NM, USA; Gombe Stream Research Centre, The Jane Goodall Institute, Kigoma, Tanzania; Kibale Chimpanzee Project, Fort Portal, Ug,,anda; Department of Human Evolutionary Biology, Harvard University, Cambridge, MA, USA; Department of Anthropology, University of New Mexico, Albuquerque, NM, USA

**Keywords:** health, aging, chimpanzees, sex differences, life history

## Abstract

**Background and Objectives:**

Understanding health and aging in one of our closest relatives, wild chimpanzees, provides key insights into the evolutionary origins of human disease risk. In humans, females often experience higher rates of disease than men despite having longer lifespans. Here we examine age- and sex- related patterns of health burdens in three communities of wild chimpanzees to investigate whether males exhibit health disadvantages, as predicted by life history trade-offs, or whether females exhibit health disadvantages in line with the health-survival paradox.

**Methodology:**

We analyzed 16 years of observational health data from the Kasekela and Mitumba communities of Gombe National Park, Tanzania, and the Kanyawara community of Kibale National Park, Uganda. We recorded clinical signs of respiratory illness, diarrhea, and injuries, along with annual cumulative health signs. We used generalized linear mixed models to examine the effects of sex and age on these measures while controlling for community differences and annual and seasonal patterns.

**Results:**

Respiratory clinical signs increased with age, but there were no sex differences. Males exhibited significantly increased likelihood of injuries and diarrhea with peaks in middle-aged years, and higher cumulative health burdens than females throughout adulthood.

**Conclusions and Implications:**

Our findings align with predictions from life history theory, suggesting that males prioritize reproductive effort over somatic maintenance, leading to greater health risks. The absence of a male–female health survival paradox in chimpanzees suggests that this pattern in humans is a derived trait, shaped by sociocultural, lifestyle, and environmental factors. These results highlight the importance of cross-species comparisons in understanding the evolution of health and aging.

## INTRODUCTION

Globally, humans are living longer than they ever have, throwing a fresh spotlight on the biological constraints on lifespan and the factors that promote healthy aging. Life expectancies, which have increased dramatically in wealthier populations [1], are often used as a proxy measure for the relative health of a population and have been the primary focus of comparative, evolutionary studies. While it is a truism that good health promotes long life, the association between longevity and healthspan is not straightforward. For example, the variation in ecology and health infrastructure across human populations produces substantial effects on early life survival but does not appear to significantly affect the rate of aging [2]. Remarkably, wealthy populations with the longest lifespans suffer dramatically higher rates of cardiovascular disease, reproductive cancers, and other debilitating age-related conditions than do preindustrial populations [3–8]. Additionally, many human populations exhibit a male–female health-survival paradox [9–11] such that men die at higher rates than women across the lifespan [12, 13], but women often experience higher rates of disease than men [14, 15]. This gender difference varies by socioecological and epidemiological context (e.g. [16, 17]) and is complicated by sociocultural processes that are entangled with gender roles in society and gender differences in provision of health care. Broader comparative data, including from closely related species, can help disentangle the complex biological, cultural, behavioral, and environmental risk factors influencing human health disparities across the life course.

Here, we examine variation in observed clinical signs of ill health in a longitudinal sample of wild East African chimpanzees (*Pan troglodytes schweinfurthii*) comprising daily surveillance of three study communities for over 15 years. Primates sit near the extreme among mammals in the slow pace of their life history traits, including long gestations, low reproductive rates, late age of weaning and reproductive maturity, and relatively long lifespans [18]. As one of humans’ two closest living evolutionary relatives, chimpanzees are genetically similar to humans, long lived (60+ years), and share multiple commonalities in biological aging with humans (e.g. neuronal aging [19]; glucocorticoid regulation [20]; social aging [21]). However, wild chimpanzees live in the absence of both the novel risk factors and protective factors that shape the contemporary human aging landscape, such as smoking, processed diets, and access to health care. Our aims are to determine: (i) how the risks of illness and injury vary across the life course in chimpanzees and (ii) how these risks contribute to sex differences in health. By applying our analysis to three communities in two different populations, we aim to determine whether the timing and magnitude of these patterns occurs reliably in chimpanzees or are variably influenced by local context. Finally, we aim to determine whether there are commonalities in these patterns between humans and chimpanzees which would suggest shared biological foundations for aging and sex differences in health.

### Aging and health in evolutionarily relevant contexts

The environmental factors shaping health and lifespan in wild chimpanzees are broadly similar to those of humans prior to the epidemiological transition, as evidenced by comparisons to contemporary foragers. Across the lifespan, both chimpanzees and foragers die primarily from trauma (violence/accidents) and from infectious diseases (e.g. respiratory and diarrheal diseases), and rarely from degenerative diseases (human foragers [22]; chimpanzees [23, 24]). When they do not directly cause death, these health insults have the potential to impact quality of life (by reducing mobility, social relationships, and the ability to meet subsistence and caregiving needs) with damage and repair costs expected to accumulate to exact a long-term toll [25, 26]. Age-related risks of illness or injury may vary through routes of exposure and biological susceptibility. Across mammals, both the very young and very old experience reduced immunocompetence, compromising the ability to combat infection [27, 28]. Older individuals may also experience changes in activity, social participation, or social status that exacerbate this risk [29, 30], or afford some compensatory protection [31–33].

### Sex differences in health in primates

The female survival advantage found in humans is mirrored in most of our nonhuman primate relatives [34]. Male and female primates pursue different activities at different life stages [35, 36], which places them at variable risk of exposure to disease vectors, including infected conspecifics, and to physical risks, such as from exertion, falls, and conflicts with other individuals (reviewed in [37]). Survival enhancing investments, such as immune function, trade-off throughout the lifespan with other fitness-promoting functions of growth and reproduction [38, 39]. Reproductive success in female mammals is closely tied to longevity, as the burden of gestation and lactation limits the number of offspring a female can produce in a lifetime [35]. Males, however, can achieve higher lifetime reproductive success by seeking additional mates and increasing mating success [40]. Several life history-derived hypotheses predict that males will sacrifice maintenance and immune function, while females prioritize those functions thereby increasing longevity [41, 42]. Thus, in contrast to the health-survival paradox detailed above, this framework predicts that females will have increased survival and health compared to males.

Only one study thus far has examined age- and sex-specific differences in multiple measures of health across the lifespan in known-aged wild primates. Alberts et al [9] compared the health trajectories of males and females in wild baboons (*Papio cynocephalus*) in Amboseli National Park, Kenya; a population with documented female survival advantage [34, 43]. This team examined body mass index, richness of gastrointestinal parasites, infection burden of whipworm parasites, and incidences of observed illness and injuries. While males exhibited an increase in incidence of observed illness with age, the authors found limited evidence for either a male or a female health advantage among the other measures.

### Assessing ill health in non-human primates

Published data from wild primates have largely recapitulated the female advantage for survival [34, 44]. However, there is a paucity of systematic studies on health measures across the lifespan from primates in the wild, where individuals are exposed to the natural range of diseases and hazards for their species. Such studies are exceedingly difficult to conduct for several reasons. First, the primates most closely related to humans, the catarrhine monkeys and apes, have long lifespans, requiring consistent decades-long research effort to capture the necessary data on known-aged individuals. Second, standardized clinical measurements of health insults or pathogen infection status have historically required direct handling and/or invasive (e.g. blood or tissue) sampling methods that are rare at most research sites due to anesthesia-associated risks and issues related to disrupting habituation and/or behavioral research [45–47]. In addition, while our ability to detect certain pathogens using non-invasively collected samples (e.g. feces and urine) has greatly expanded in the past 20 years (e.g. [45, 48]), these methods can be costly and logistically difficult to implement for routine population-wide screening. Furthermore, not all diseases of concern have had the necessary diagnostic tests developed, and the spectrum of possible pathogens in a population is rarely known. Given these challenges, researchers and protected area managers often prioritize a form of syndromic surveillance, which entails the collection of pre-diagnosis health data that signals a case or outbreak [49]. This includes the collection of clinical signs, which are observed indicators of potential medical conditions used by both human and veterinary doctors to assist with diagnosis [50].

### Wild chimpanzee life history and socioecology

Chimpanzees (*Pan troglodytes*) are one of human’s two closest living relatives and have been studied at multiple long-term study sites across Africa. They are also the longest-living wild primate with maximum lifespans reported of 66 years for females and 57 years for males [51, 52], with males experiencing higher mortality rates across the adult lifespan (reviewed in [53]). Chimpanzees exhibit a number of sex differences in behavior that may affect health status at various points of the life course. The chimpanzee mating system is promiscuous, and direct parental care is provided solely by mothers [54]. Maternal investment is extensive: offspring are nutritionally dependent on their mother until weaning between the ages of 3 and 5 years [55], but remain behaviorally dependent (i.e. continually traveling and socializing with) through the juvenile years, many until the age of 10 [56]. Reproductive effort for females is therefore persistent across adulthood, whereas male reproductive effort peaks in early- to mid-adulthood (~ages 15–35) and declines at older ages [52, 57]. Chimpanzee females are socially disadvantaged, in that all males are normally dominant to all females [54]. In Eastern chimpanzees (*P. troglodytes schweinfurthii*), females are less gregarious and typically range and feed in small overlapping core areas while males are more gregarious and range broadly throughout the territory [58–60], potentially increasing their pathogen exposure. Male chimpanzees also participate in substantially more direct physical aggression than females, both within communities during sexual coercion and competition for dominance status and mates, and between communities during cooperative territorial defense [61–63]. Although less common, female aggression can also be severe, including infanticides and attacks against newly immigrating females [64, 65].

Chimpanzee health parameters, such as clinical signs or parasitic infections, have been studied at multiple sites, but these studies have rarely had sufficient sample size to conduct age-controlled analyses. Thus far, they have yielded inconclusive evidence for sex differences in health. A previous study of clinical signs at Gombe National Park, Tanzania, reported higher rates of wounding among males but no sex differences in other measures, but this study only compared broad adult and immature age categories [66]. These prior studies do point to respiratory illness is a dominant cause of morbidity and mortality in wild chimpanzees [67–69]. Whereas many community-wide lethal ‘epidemics’ of respiratory illness have been linked to viruses of human origin [70, 71] and/or secondary bacterial infections [68, 72, 73], episodic low-grade illness is observed more commonly, and its etiology is not yet understood [66, 67]. Injuries, particularly from conspecific aggression and falls, are a frequent cause of mortality [24, 67] and when not fatal, could present a persistent challenge for chimpanzees to resist infection and move about the forest to meet their daily caloric needs. While previous studies on chimpanzee injuries have focused on skeletal trauma (e.g. [74]), which captures the most severe injuries and is limited to small sample sizes of recovered skeletons, here we examine observable injuries in living individuals. Diarrhea is also an important health indicator, as wild chimpanzees exhibit frequent parasitic and viral infections that affect the gut [48, 75–79]. Other clinical signs, including wasting, vomiting, lethargy, and skin conditions are far less common and often secondary to one of the above causes. Thus, observations of respiratory signs, diarrhea, and injuries, while non-specific, capture a broad range of health insults affecting wild chimpanzees.

In this contribution, we used long-term data on three communities of wild chimpanzees from two different study populations to examine clinical signs of health across the lifespan. We compiled health and demographic data from the Kasekela and Mitumba communities of Gombe National Park, Tanzania, and the Kanyawara community of Kibale National Park, Uganda, to generate the largest longitudinal and comparative health dataset on wild chimpanzees to date. Specifically, we examined clinical signs of respiratory illness, diarrhea, and injuries, as well as an annual cumulative measure of all three categories. We used these data to characterize age-related patterns of health insults across the lifespan, and to investigate whether males exhibit health disadvantages, as predicted by life history trade-offs, or whether females exhibit health disadvantages in line with the health-survival paradox.

## METHODS

### Study sites and long-term data collection

Gombe National Park (land area = 35 km^2^) is located on the western border of Tanzania and is the site of The Jane Goodall Institute’s Gombe Stream Research Centre. Our study focused on the two habituated chimpanzee communities in the park, Kasekela and Mitumba. The Kasekela community was habituated in the early 1960s and full-day focal follows on individual chimpanzees commenced in 1973. Habituation of the Mitumba community began in the mid-1980s and full-day focal follows commenced in the mid-1990s [80]. During these follows, a team of two trained observers records group composition and location every 15 minutes, continuous data on feeding behavior, and all-occurrence data social interactions. Observers attempt to follow the majority of adult chimpanzees in each community at least once per month as part of this behavioral record. Additionally, in the larger Kasekela community, follows on family groups (mother, infant, and next oldest offspring) are conducted with the aim of targeting each family once per month (see [81] for a detailed description). Standardized data on the presence or absence of observable clinical signs has been collected as part of these daily behavioral observations since early 2004 (see [66, 82] for a detailed description of data collection protocols). For this study, we analyzed data from April of 2004 through December of 2019 (data collection for all communities was impacted for much of 2020 and beyond due to the COVID-19 pandemic). During this time, the Kasekela community size ranged from 49 to 62 individuals, comprised of 9–14 adult (12 years of age or older) males, 19–27 adult females, and 16–26 immature individuals. The dataset for Kasekela includes 5205 follows and an average of 8.7 hours (s.d. 3.4 hours) of observation per follow. The Mitumba community size ranged from 21 to 30 individuals, comprised of 2–6 adult males, 8–12 adult females, and 9–15 immature individuals. The dataset for Mitumba includes 4614 follows and an average of 8.1 hours (s.d. 3.5 hours) of observation per follow.

Members of the Kibale Chimpanzee Project study the Kanyawara community of chimpanzees in Kibale National Park, Uganda. The long-term study of the Kanyawara chimpanzees was established by Wrangham in 1987 [83], and thereafter chimpanzees have been followed on a near-daily basis. Both focal and group data are collected simultaneously by a team of trained observers, where a pair of researchers selects a focal individual to follow for the day recording all focal behavior every minute, while a second researcher records group composition and location every minute and all-occurrence social interactions. Ideally, each chimpanzee in the community is the subject of a focal follow every month and is followed from nest-to-nest. Observers collaborate to record any occurrence of clinical signs for all individuals observed that day [67]. For this study, we analyzed data from January of 2004 through December of 2019, which includes 7301 follows and an average of 9.8 hours (s.d. 3.5 hrs) of observation per follow (n.b., multiple follows are sometimes performed on the same day on different chimpanzee subgroups). During this time, the Kanyawara community size ranged from 41–56 chimpanzees, including 9–13 adult males, 13–18 adult females, and 18–28 immature individuals.

Both study sites maintain long-term demographic records of individual chimpanzee birthdates and sex and have contributed to the Primate Life Histories Database and Working Group, an effort to standardize demographic data across primate populations [84]. Thus, robust criteria for age estimation have been established and are consistent across these study populations. Over 70% of individuals in the study have ages known within one month (because they were born since observations began) or within one year (because they were first encountered at a young age). See Table 1 for numbers of individuals, chimpanzee months and averaged observation days per month in the final sample by community and sex, as well as age ranges. All animal procedures were observational, non-invasive and approved by the Institutional Animal Care and Use Committees of Emory University, University of New Mexico and Tufts University.

**Table 1:** Numbers of individuals, chimpanzee months and mean observation days per month in the final sample by community and sex with accompanying age ranges.

Community	Sex	# Individuals	# Chimpanzee months	Mean observation days per month	Age range (yrs)
Mitumba (MT)	F	25	1653	12.8	0.2–46.5
	M	18	959	11.6	0.2–30.5
Kasekela (KK)	F	58	2989	10.5	0.0–56.8
	M	41	2175	11.9	0.0–42.7
Kanyawara (KY)	F	53	4323	13.3	0.0–63.5
	M	39	4055	13.8	0.0–58.2

### Clinical signs

The clinical sign definitions used at both sites are largely identical, following the definitions reported in Lonsdorf et al [66]. To harmonize the datasets, EVL and MET reviewed any questionable cases to ensure consistent assignment of clinical signs. We focused on three categories of clinical signs that are the most conspicuous to observers and represent the vast majority of signs recorded. These were defined as follows:

1) Respiratory—presence of (i) a ‘wet’ or ‘productive’ cough; (ii) coughing and sneezing on the same day; (iii) a runny nose; or (iv) frequent or severe coughing or sneezing (e.g. ‘coughing very badly’ or ‘coughing all day’).
2) Injuries—observed or suspected lesion to any tissues of body caused by trauma or disease. Included in this category are observations of ruptured, abraded, or swollen skin and impaired use of limbs or appendages, indicating an internal injury. Human-caused snare injuries are frequent at Kanyawara and absent at Gombe, so we excluded these from the dataset.
3) Diarrhea—presence of watery stools that no longer retain shape or consistency.

Other clinical signs are collected at each site but are either too infrequent for analyses (skin rashes and other skin conditions) or were not collected in all communities (e.g. categorical assessment of body condition has only been collected for the whole study period at Gombe).

Given that chimpanzees are characterized by high fission-fusion dynamics and can range over large areas [85, 86], individuals cannot be observed every day. Therefore, we followed prior studies of wild chimpanzee clinical signs [66, 67] and coded a dichotomous variable for each individual in each calendar month of the study (i.e. chimpanzee-month) indicating whether or not the individual was observed on at least one day with a particular clinical sign (respiratory, injury, diarrhea). This approach also reduces problems of non-independence, such as when the same clinical sign was observed several times, or when it is not clear that two successive observations were indicative of the same event. We collected nearly 16 years of data from each community (2004–2019 complete for Kanyawara, April 2004–2019 for Kasekela and Mitumba) for respiratory and diarrhea signs. For injuries, we collected 15.75 years of data from Kasekela and Mitumba (April 2004–2019), and 14 years from Kanyawara (2006–2019), where standardized injury data collection commenced in 2006. We excluded individuals that were observed for less than one year of the study (e.g. infants that did not survive the first year of life, and individuals in the process of transferring communities that only appeared for a few months).

### Statistical analyses

We conducted all analyses in R version 4.4.1 [87] and RStudio version 2024.09.0.375 [88] using the following packages: glmmTMB version 1.1.0 [89], DHARMA version 0.4.6 [90], car version 3.1–2 [91], and performance version 0.12.2 [92] packages. For each individual clinical sign, we analyzed the presence/absence (1/0) of that sign in each chimpanzee in each month using generalized linear mixed models (GLMMs) with a binomial error distribution and a logit link function. For these analyses, we calculated chimpanzee age (in years) on the date of the first health observation in a given month (whether ill or healthy) and then z-score transformed for analyses. Because younger and older individuals may be at higher risk for ill-health, we initially included both a linear and quadratic term for age. We also included a fixed effect for community in all models to control for potential community differences. To control for potential seasonal influences, we converted each calendar month into circular variables comprised of two sine-plus-cosine functions [93] with annual and semiannual periodicities. Given that there is variation in the number of observation days among individuals, which could influence the probability of observing a clinical sign, we included an offset term of the log of total days observed for each individual in each month. All models included individual as a random intercept to address repeated and uneven sampling of individuals. We also included calendar year as a random intercept, with community as a random slope, to account for differences in likelihood between communities in a particular year due to outbreaks of illness [24] and/or violence [94].

We started with a full model for each clinical sign comprised of the predictors described above as well as the following interaction terms: (i) sex and age, to test for sex-differentiated patterns by age, (ii) community and age, to examine whether age-related patterns are consistent across communities, and (iii) sex and community, to examine whether sex-differentiated patterns are consistent across communities. We tested interaction terms with *P* < 0.10 in the full model against models excluding the interactions using likelihood ratio tests implemented in the anova function of the car package; we excluded those did not significantly improve model fit.

To examine correlates of cumulative ill-health, we calculated a measure of cumulative clinical signs in each age year for each chimpanzee. That is, we summed the number of unique clinical signs (respiratory, injury, diarrhea) in each month (possible range 0–3) for the entire age year (possible range 0–36 for 12 months) for each individual. To analyze cumulative clinical signs in an age year, we used a GLMM with a Poisson error distribution and log link function. As with the individual signs, we began with a full model similar to those described above; however, we did not include terms for seasonality here since an individual’s full age year would encompass all seasons. In addition, we did not include random effects for calendar year since each individual’s age year can encompass more than one calendar year.

For all models, we used functions in the DHARMa package to confirm that there were no influential outliers (testOutliers), that models were not overdispered (testDispersion), and to examine zero inflation (testZeroInflation). We used the check_collinearity function in the performance package to examine variance inflation factors of main effects and confirm no unexpected collinearity (the four seasonal terms are expected to be correlated). To assess the stability of our models, we compared estimates derived from models with each level of the random effect (ID and/or year) dropped one at a time. These assessments revealed no influential levels. We used the anova function in the car package to compare final models to a null model that contained only random effects and the offset term.

## RESULTS

### Respiratory clinical signs

**Figure 1:**
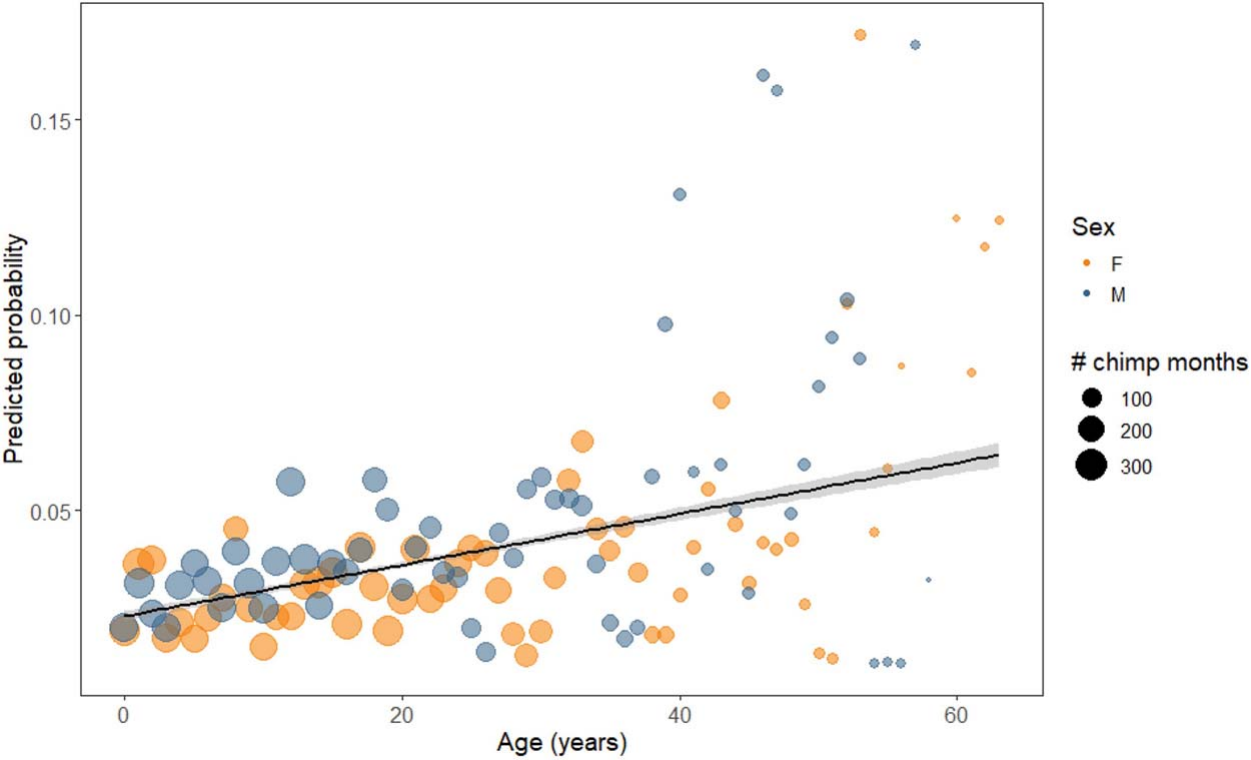
Model predicted probability of respiratory signs across age. Each point represents an age year in each sex, scaled in size according to the number of chimp months comprising that age year. The fitted line represents glm smooth with 95% confidence intervals.

There were 562/16154 chimpanzee months (3.5%) with respiratory clinical signs. The likelihood of respiratory clinical signs increased with age, and there were significant seasonal effects (see Table 2, Fig. 1). However, there were no significant sex differences.

**Table 2:** Results of a generalized linear mixed model examining predictors of respiratory clinical signs.

**Term**	**Estimate**	**SE**	***Z***	***P***
Intercept	−6.493	0.292	−22.2	**<0.001**
Sex (M)	0.129	0.108	1.19	0.234
Age	35.79	6.447	5.55	**<0.001**
Age^2^	2.806	5.952	0.471	0.637
Community (KY)	0.282	0.425	0.664	0.507
Community (MT)	0.074	0.476	0.156	0.876
Sine annual	6.799	0.759	8.95	**<0.001**
Cosine annual	7.593	1.852	4.1	**<0.001**
Sine semiannual	−7.734	0.905	−8.55	**<0.001**
Cosine semiannual	−6.449	1.753	−3.69	**<0.001**

Significance (*P* < 0.05) is indicated in bold. The reference category for all sex-based variables is female. The reference category for community is KK; KY indicates Kanyawara and MT indicates Mitumba. The final model provided significantly better fit than the null model (χ2 = 127.15, df = 9, *P* < 0.001).

### Injuries

**Figure 2:**
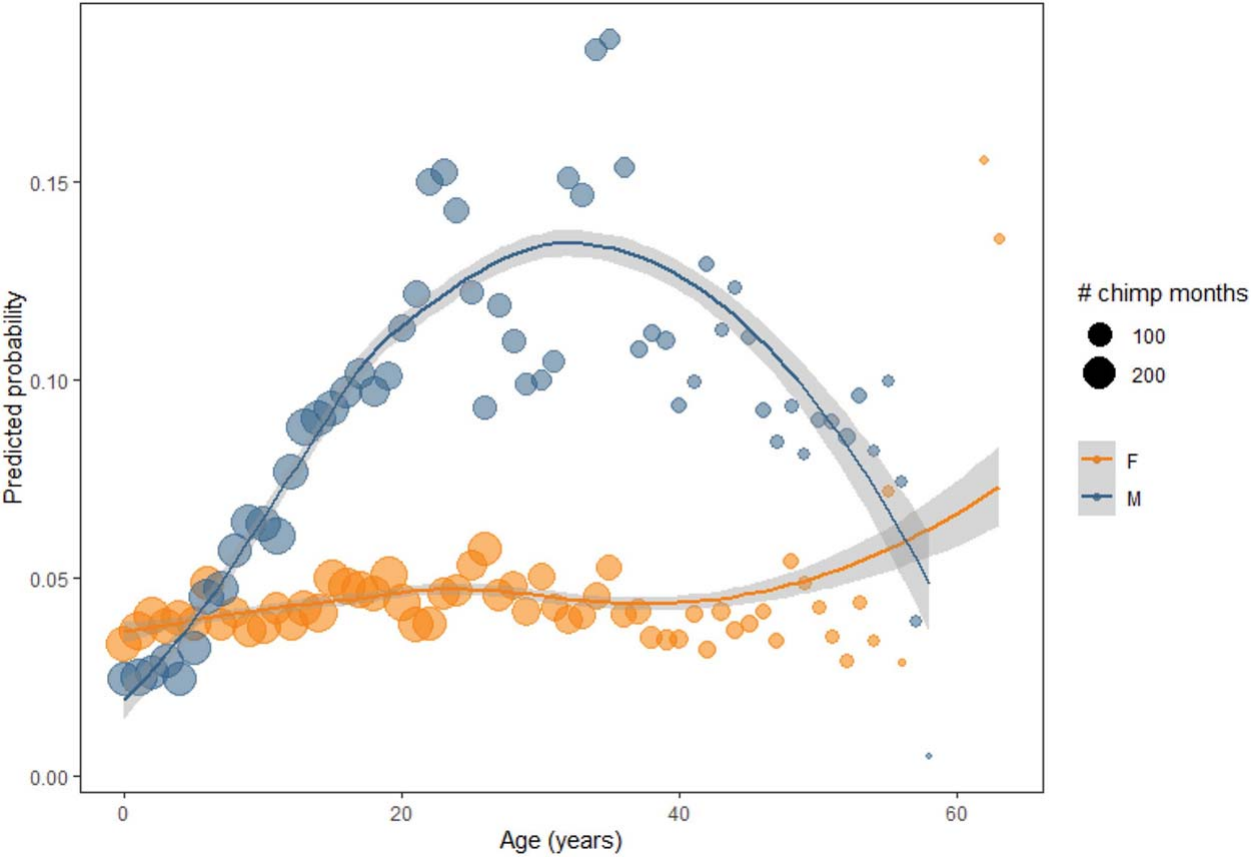
Model predicted probabilities of injury, separately by sex. Each point represents an age year, scaled in size according to the number of chimp months comprising that age year. Fitted line represents a loess smooth with 95% confidence intervals.

**Figure 3:**
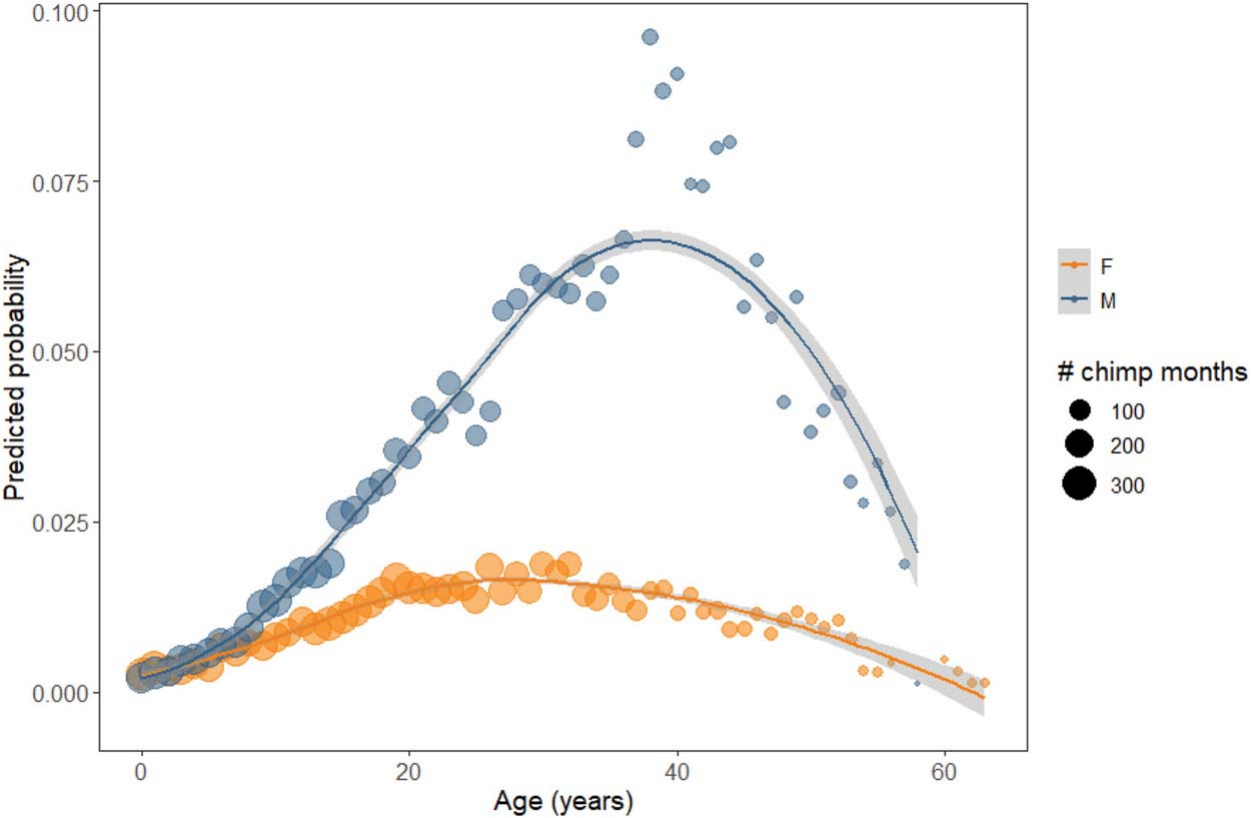
Model predicted probabilities of diarrhea, separately by sex. Each point represents an age year, scaled in size according to the number of chimp months comprising that age year. Fitted line represents a loess smooth with 95% confidence intervals.

There were 923/15277 chimpanzee months (6.0%) with injuries. We found a significant quadratic age by sex interaction on the likelihood of injuries, indicating that the curvilinear age effect depends on sex. Figure 3 shows that males have increased likelihood of injuries in middle age while females did not. Rather, females exhibited a more consistent likelihood of injuries across the lifespan, except for one elderly female that was persistently injured towards the end of her life. Males were more likely than females to be observed with injuries at most ages (see Table 3, Fig. 2). While individuals in Kanyawara were more likely than those in Kasekela and Mitumba to be observed with injuries, the sex difference was consistent across communities. There were no significant seasonal effects.

**Table 3:** Results of a generalized linear mixed model examining predictors of injury.

**Term**	**Estimate**	**SE**	***Z***	***P***
Intercept	−6.061	0.189	−32.11	**<0.001**
Sex (M)	0.482	0.128	3.76	**<0.001**
Age	24.25	10.16	2.39	**0.017**
Age^2^	−4.063	8.893	−0.46	0.647
Community (KY)	0.489	0.202	2.43	**0.015**
Community (MT)	−0.078	0.314	−0.25	0.804
Sine annual	0.304	0.633	0.48	0.631
Cosine annual	−0.482	1.391	−0.35	0.729
Sine semiannual	−0.266	0.762	−0.35	0.727
Cosine semiannual	0.510	1.30	0.39	0.695
Sex (M)*Age	47.17	14.49	3.25	**0.001**
Sex (M)*Age^2^	−41.95	12.96	−3.24	**0.001**

Significance (*P* < 0.05) is indicated in bold. The reference category for all sex-based variables is female. The reference category for community is KK; KY indicates Kanyawara and MT indicates Mitumba. The final model provided significantly better fit than the null model (χ2 = 90.77, df = 11, *P* < 0.001).

### Diarrhea

There were 296/16154 chimpanzee months (1.8%) with diarrhea. During model fitting, inclusion of the random slope for community in each calendar year resulted in convergence problems and was therefore excluded; instead, we included a random intercept for year. The final model included significant quadratic age effects on the likelihood of diarrhea, such that males and females both showed increased likelihood of diarrhea in middle age. In addition, males were significantly more likely to be observed with diarrhea at most ages (see Table 4, Fig. 3). There were also significant seasonal effects, and community differences, with individuals in Kanyawara and Mitumba less likely than those in Kasekela to be observed with diarrhea. However, the sex difference was shared by all communities.

**Table 4:** Results of a generalized linear mixed model examining predictors of diarrhea.

**Term**	**Estimate**	**SE**	***Z***	***P***
Intercept	−6.661	0.239	−27.8	**<0.001**
Sex (M)	0.565	0.193	2.93	**0.003**
Age	43.82	17.95	2.44	**0.015**
Age^2^	−47.48	18.52	−2.56	**0.010**
Community (KY)	−0.639	0.204	−3.13	**0.002**
Community (MT)	−0.466	0.261	−1.78	*0.075*
Sine annual	0.561	1.115	0.50	0.615
Cosine annual	9.107	2.369	3.84	**<0.001**
Sine semiannual	−1.951	1.352	−1.44	0.149
Cosine semiannual	−8.939	2.201	−4.06	**<0.001**
Sex (M)*Age	71.41	24.14	2.96	**0.003**
Sex (M)*Age^2^	6.975	23.42	0.30	0.766

Significance (*P* < 0.05) is indicated in bold; (*P* < 0.10) is indicated in italics. The reference category for all sex-based variables is female. The reference category for community is KK; KY indicates Kanyawara and MT indicates Mitumba. The final model provided significantly better fit than the null model (*χ*2 = 114.2, df = 11, *P* < 0.001).

### Cumulative annual signs

**Figure 4:**
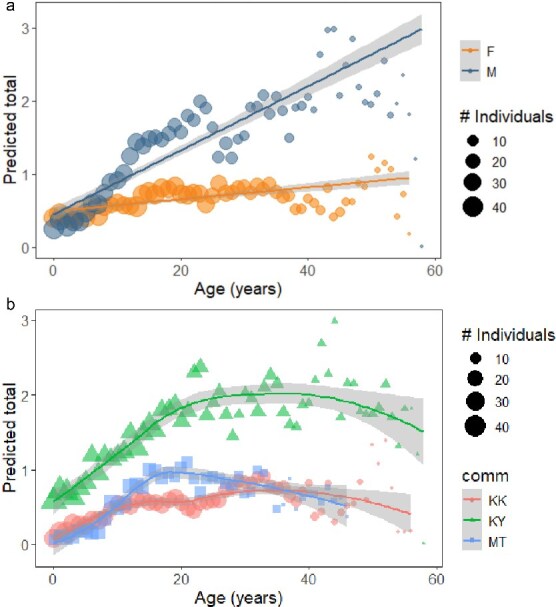
(a) Model predicted counts of annual clinical signs, separately by sex. Each point represents an age year, scaled in size according to the number of individuals comprising that age year. Fitted line represents a glm smooth with 95% confidence intervals. (b) Model predicted counts of annual clinical signs, separately by community. Each point represents an age year, scaled in size according to the number of individuals comprising that age year. Fitted line represents a loess smooth with 95% confidence intervals.

Our initial dataset encompassed 2028 chimp years, and the cumulative number of signs observed annually ranged from 0 to 10. Diagnostic testing on the final model detected the presence of outliers, which led to the removal of a single female over 60 years old in Kanyawara. This removal resolved the issue with no substantive changes to the model results. The final reduced dataset encompassed 2026 chimpanzee years with the same range of annual cumulative signs (0–10). Diagnostic testing also indicated slight zero inflation, so we included a zero-inflation term in the final model [95]. Males exhibited significantly more signs than females, beginning in the early juvenile period, and this difference increased significantly with age (see Table 5, Fig. 4a). In addition, there were significant quadratic age effects by community, such that the curvilinear age effect differs significantly for Kanyawara when compared to Kasekela, and marginally for Mitumba when compared to Kasekela (see Table 5, Fig. 4b).

**Table 5:** Results of a generalized linear mixed model examining predictors of cumulative annual clinical signs.

**Term**	**Estimate**	**SE**	***Z***	***P***
Intercept	−5.024	0.085	−59.16	**<0.001**
Sex (M)	0.294	0.078	3.79	**<0.001**
Age	4.733	3.392	1.40	0.163
Age^2^	0.995	3.379	0.29	0.768
Community (KY)	0.216	0.087	2.47	**0.013**
Community (MT)	−0.142	0.132	−1.08	0.281
Sex (M)*Age	13.09	3.492	3.75	**<0.001**
Sex (M)*Age^2^	−1.994	3.270	−0.61	0.542
Age*Community (KY)	4.890	3.791	1.29	0.197
Age^2^*Community (KY)	−9.071	3.992	−2.27	**0.023**
Age*Community (MT)	−2.946	6.262	−0.47	0.638
Age^2^*Community (MT)	−12.35	7.046	−1.75	*0.079*

Significance (*P* < 0.05) is indicated in bold; (*P* < 0.10) is indicated in italics. The reference category for all sex-based variables is female. The reference category for community is KK; KY indicates Kanyawara and MT indicates Mitumba. The final model provided significantly better fit than the null model (χ2 = 108.71, df = 11, *P* < 0.001).

## DISCUSSION

Our findings provide new insights into health burdens across the lifespan in wild chimpanzees, highlighting both parallel and divergent patterns when compared to humans. Across the three types of clinical signs that we most commonly observed in these chimpanzees, only respiratory signs increased in a progressive fashion with age, consistent with age-related immunosenescence. This pattern did not differ between males and females. The prevalence of diarrhea and injuries were both higher in males than in females, with sex differences emerging in the early juvenile period and peaking in middle adulthood. While absolute rates of clinical signs differed across communities, the age and sex patterns were consistent across the three communities. Taken together, these data suggest that male chimpanzees experience a chronically higher burden of ill health across the lifespan compared with females, in line with hypothesized life history trade-offs with reproductive effort. However, these sex differences were not consistent with accelerated male senescence but instead correspond to successive peaks in risk from different causes across the adult lifespan.

### Respiratory

Respiratory disease is one of the most significant causes of morbidity and mortality for wild chimpanzees [24, 67, 70, 96]. In this study, the likelihood of respiratory clinical signs increased with age, likely reflecting increased susceptibility due to age-related immunosenescence. While susceptibility to respiratory illness in humans varies by pathogen, prevalence tends to be highest in young and old individuals [97]. The severity of infection for many respiratory viruses varies with sex according to life stage; males tend to be more susceptible to severe outcomes at older and younger ages, while females are at greater risk for severe outcomes during reproductive years [97]. Our results parallel those in humans with regards to increased likelihood of respiratory clinical signs with age. However, respiratory signs were rarely observed in young individuals (< 10 years) and there was no significant age-sex pattern. Rather, respiratory illness may be a reliable indicator of immunosenescence in wild chimpanzees that is less sensitive to sex differences in life history strategies.

Notably, we found no differences between males and females in the age-specific likelihood of being observed with respiratory signs. These results contrast with predictions about sex differences in immune function derived from life history theory, which predict higher male susceptibility due to higher testosterone, costlier behaviors, increased gregariousness, and reduced investment in maintaining the immune system [35, 37]. Prior research on wild chimpanzees reported inconsistent evidence of sex differences, ranging from no sex difference (Gombe [66]; single outbreak at Ngogo [70]) to female bias (single outbreak at Kanyawara [70]) to distinct aging patterns in the two sexes (Kanyawara [67]). It is important to note that respiratory signs may be caused by myriad of identified (e.g. *Streptococcus pneumoniae* and paramyxoviruses [68]; human rhinovirus C [67]; metapneumovirus and human respirovirus 3 [70]) and as-yet unidentified pathogens, which may variably affect the age and sex patterns of illness. The present study benefits from the combination of a deep longitudinal sample, continuous measures of age, and controls for the significant seasonal influence on respiratory signs. As a result, this study spans numerous outbreaks and respiratory infections and therefore represents long-term patterns of sex differences. Future studies should consider pathogen-specific effects on each sex.

### Injuries

Injuries were the most frequently observed clinical sign in our dataset, with significant age-sex interactions. Injuries in wild primates may arise from various causes, including accidental falls [85], interspecific aggression and predation [98], and most commonly, conspecific aggression [99, 100]. Males were significantly more likely to be observed with injuries across all life stages except infancy, which corresponds with higher rates of male aggression [63] and more frequent participation in risky behavior such as hunting other primates [101–103] and territorial boundary patrols [103]. This male-bias for injuries is consistent with previous work in the Gombe chimpanzees [66] and is generally consistent with patterns of sex differences in skeletal trauma [104], although these sex differences are less pronounced [74, 105]. Across most wild primates that have been studied (blue monkeys (*Cercopithecus mitis*) [106], olive baboons (*Papio anubis*) [99], Nepal gray langurs (*Semnopithecus schistaceus*) and Phayre’s leaf monkeys (*Trachypithecus phayrei crepusculus*) [107]; rhesus macaques (*Macaca mulatta*) [108], and humans [109, 110], males are more frequently observed with injuries.

In male chimpanzees, the likelihood of being observed with injuries increased from infancy to the mid-30s before beginning to decline, while females had a more consistent and low likelihood of injuries over the life course. Notably, our findings contrast with the hypothesis that weaker physical condition and lower social status lead to an increased risk of injuries with advancing age. Instead, the peak in risk of injuries in males corresponds to the years of highest status and reproductive effort and likely reflects injuries due to competition over dominance and reproductive opportunities [57]. For older males, the reduced likelihood of injury may reflect changing reproductive strategies [52] and/or changes in patterns of social engagement with age. Older male chimpanzees have lower rates of aggression, more equitable relationships, and spend less time in social groups [21]. Female chimpanzees increase in status with age [111] and become more socially isolated [112], which may buffer them from injuries as they age. We identified significant community differences, with a higher likelihood of injury at Kanyawara, but the broad age and sex patterns were replicated at all three communities. It should be noted that we observed a relatively low rate of injuries among males at Mitumba, though there is a high rate of lethal male aggression in this community [113]. This may also explain why the age peak in injury at Mitumba is shifted toward an earlier part of adulthood, as adult male life expectancy in this community has recently been poor.

### Diarrhea

Diarrhea was the least prevalent clinical sign in this dataset, occurring in < 2% of chimpanzee months. The likelihood of being observed with diarrhea was higher in the middle-age years for both males and females when compared to young or old individuals, and males were consistently more likely to be observed with diarrhea across adulthood. Previous reports at Gombe found no sex differences in the likelihood of diarrhea [66], but those studies did not control for age differences among the adults.

Diarrhea in wild chimpanzees likely results from diverse causes, including gastrointestinal pathogens [77, 114], dietary changes and/or sensitivities [115], and stress [116]. It is also possible that recurrent enteric infections reflect adaptive immune trade-offs. Frequent exposure to gut pathogens may generate cross-reactive immune responses that provide protection against more serious systemic or respiratory infections, making diarrhea a visible but potentially acceptable cost of broader immune defense [117]. Alternatively, diarrhea has recently been shown to be an active and deliberate host defense mechanism to eliminate pathogens, rather than a mere symptom of illness [118, 119].

Both males and females showed a quadratic age pattern, with diarrhea more common in middle-aged than in the youngest or oldest individuals. These patterns suggest a more complicated explanation than just immunosenescence, though it is clear that immunosenescence plays a role in susceptibility to gastrointestinal infection. For example, in prior studies in Kibale, gastrointestinal viral richness and viral loads increased linearly with age in males, though not in females [114] and shedding of at least some parasites increased with age in females [120]. However, mirroring the pattern we observed here, Gillespie et al [77] found that prime age individuals at Gombe had higher prevalence of most pathogenic parasites compared to younger and older individuals. It is likely that these sex and age-related patterns reflect the interplay between chimpanzee behavior and energetic tradeoffs with the immune system. For example, male chimpanzees hunt vertebrate prey at higher rates than females at all three of the communities studied here [121] and hunting probability peaks in prime age males [122]; hunting is both energetically expensive and may expose hunters to gastrointestinal pathogens. Additionally, a small study of male chimpanzees at Ngogo reported a significant positive association between parasite richness detected in feces and both dominance rank and testosterone [123], which peak in early adulthood [124]. However, the pattern of diarrhea we observed here was shifted toward older ages for males than the pattern for injuries and did not correspond to the years of high reproductive effort. Instead, the age patterns we observed for both sexes more closely resemble age-related patterns of social integration for chimpanzees, which peak in early adulthood for females and in middle adulthood for males [112]. Gastrointestinal parasite infection risk has been shown to be positively associated with higher measures of social integration in chimpanzees and other primates (reviewed in [125]). We suspect that the likelihood of diarrheal clinical signs is driven by a complex combination of social and dietary exposures, energetic tradeoffs, and immunosenescence, and that declining gregariousness at late ages may offset an individual’s reduced ability to resist parasites with age.

Our results contrast with those for humans, for which diarrheal illnesses are significantly more prevalent in children under five years of age and show no consistent sex differences [126, 127]. The high incidence in children has been presumed to reflect their immature immune systems, though this was not a pattern shared by infant chimpanzees. We acknowledge that diarrhea in infant chimpanzees may be more difficult to observe than among adults, especially in the first two years when infants are clinging to their mothers for large proportions of time. However, both projects included prolonged focal observations on infants using a comprehensive health checklist, yet observations of diarrhea in young chimpanzees were still exceedingly rare. Common risk factors for diarrhea in humans include unsafe drinking water and poor sanitation [127], which may be less risky for chimpanzees living at low densities and where most water intake is via consumption of fruit [128]. Taken together, these results suggest divergent age-sex patterns of diarrheal burden in humans and chimpanzees that point to different etiologies.

### Cumulative annual signs

Our analysis of cumulative health signs showed that males exhibited significantly higher annual health burdens than females throughout adulthood, and the difference increased as they aged. The sex difference emerged after infancy, and males had more than twice the rate of poor health than females through most of the adult years. As our sign-specific analyses indicate, the persistence of this health disparity was driven not by one type of health insult, but by different insults arising at different phases of the adult lifespan. For males, a peak in injuries in early to mid-adulthood was followed by a peak in diarrhea in the early past-prime period; just as diarrhea rates began to decline in later adulthood, males experienced their highest risk of respiratory illness. The community differences in cumulative health signs largely reflect the higher likelihood of injuries at Kanyawara. While our methods were highly comparable, individual chimpanzees were observed slightly more often at Kanyawara (see Table 1), increasing the probability of observing an injury before it healed.

### Implications for evolutionary medicine

Our findings align with predictions derived from evolutionary life history theory, which predict that males prioritize costly and risky reproductive effort over somatic maintenance [35–37, 41]. Our data are inconsistent with the male–female health-survival paradox observed in many human populations. Together with the findings in baboons [9], our results suggest that the male–female health-survival paradox reported in humans is either a derived feature of the human species or a novel byproduct of evolutionary mismatch in industrialized environments. However, given the overall scarcity of comparative primate morbidity data, additional long-term datasets from different primate taxa will be critical for furthering our understanding of these patterns.

Sex differences in human health result from a combination of sociocultural factors, gender disparities, and environmental and lifestyle factors that interact with evolved biological processes. There are several viable explanations for the differences between the health patterns reported in humans and those we report here for wild chimpanzees. Methodological differences could play a role as much of the evidence from humans derives from data types not available in our non-invasive study, including verbal self-reports and direct physical examinations. Thus, our methods may underrepresent diseases known to disproportionately affect women, such as autoimmune disorders [129], Alzheimer’s disease [130], and chronic pain [131]. On the other hand, these diseases feature prominently among hypothesized evolutionary ‘mismatch’ diseases that have increased rapidly in prevalence with affluent, post-industrial lifestyles [132]. This mismatch results in contradictory outcomes from the common physiological sex differences shared broadly across species. Males generally exhibit less robust immune responses to respiratory viruses [97] and bacterial infections [133] than females (except during pregnancy); these differences are theorized to be linked to the immunosuppressive effects of testosterone and pro-inflammatory effects of estrogen in both humans and other mammals (reviewed in [37, 133]). However, the generally stronger immune response of females may come at a cost of longer-term autoimmune and inflammatory conditions under particular environmental conditions [37, 97].

More generally, the available evidence indicates that the major burden of morbidity and mortality in wild chimpanzees, as in pre-industrial human populations, is from infectious disease and trauma, whereas chronic non-communicable diseases are by far the largest health burden in populations from which evidence for the male–female health-survival paradox has been based [5, 134, 135]. Additionally, female-biased morbidity in humans is most obvious after the menopausal transition and has been linked to decreases in estrogen [136]. While chimpanzees can live long enough to experience menopause [137], this is rare and would not have affected any females in our sample. Finally, while male chimpanzees are physically and socially dominant to females, this may not be as consequential for health as gender inequalities in many human societies. Whereas human females often engage in more health-seeking behaviors, potentially leading to higher rates of diagnosis (e.g. respiratory illness [138]), women’s health is also demonstrably impacted by restrictions on economic, reproductive, and political freedoms in many societies, including access to adequate health care and nutrition and protection from violence (e.g. [139]).

A final consideration that may affect apparent sex differences in health is the influence of mortality selection, whereby weaker individuals succumb to early mortality leaving a relatively robust aging cohort [140]. For example, if males disproportionately fall victim to early mortality, then the males remaining at late ages may represent a more resilient subpopulation than the females. While we certainly cannot rule out a general impact of mortality selection in our sample, such an effect could not explain our overall findings, since male-biased mortality did not produce a female health disadvantage at later ages. Nevertheless, it is notable that compared with wild chimpanzees, the human populations from which most comparative clinical data arise are relatively buffered against high rates of early mortality, and this difference is likely to shape health trajectories by age, and potentially by sex.

We also identified variation in how aging affects the prevalence of the different clinical signs in chimpanzees, revealing key contrasts with human patterns. Most notably, clinical signs are relative rare among immature chimpanzees. And while the effects of immunosenescence clearly shape the health of older individuals in these populations exposed to a wide range of pathogens, our data provide evidence that older individuals are not uniformly at higher risk. Importantly, changes in behavior may be successful at reducing exposure to some health insults at older ages, when individuals would otherwise be most vulnerable.

Without the ability to directly examine our subjects or conduct disease-specific diagnoses, we are limited in our ability to isolate the mechanisms underlying sex and age-related differences in health among chimpanzees. While more work is clearly needed and will be possible as new non-invasive tools are developed, this application of a comparative, evolutionary approach establishes an important new foundation to evaluate the evolutionary and ecological factors shaping health disparities across the lifespan.

## Data Availability

The data underlying this article are publicly available at this URL: https://doi.org/10.7910/DVN/TLGWM0

## References

[1] Vaupel JW, Villavicencio F, Bergeron-Boucher MP. Demographic perspectives on the rise of longevity. *Proc Natl Acad Sci USA* 2021;118:e2019536118. 10.1073/pnas.2019536118 33571137 PMC7936303

[2] Colchero F, Aburto JM, Archie EA et al. The long lives of primates and the “invariant rate of ageing” hypothesis. *Nat Commun* 2021;12:3666. 10.1038/s41467-021-23894-3 34135334 PMC8209124

[3] Alvarado BE, Harper S, Platt RW et al. Would achieving healthy people 2010’s targets reduce both population levels and social disparities in heart disease? *Circ Cardiovasc Qual Outcomes* 2009;2:598–606. 10.1161/CIRCOUTCOMES.109.884601 20031898

[4] Berenbaum F, Wallace IJ, Lieberman DE et al. Modern-day environmental factors in the pathogenesis of osteoarthritis. *Nat Rev Rheumatol* 2018;14:674–81. 10.1038/s41584-018-0073-x 30209413

[5] Gurven MD, Lieberman DE. WEIRD bodies: mismatch, medicine and missing diversity. *Evol Hum Behav* 2020;41:330–40. 10.1016/j.evolhumbehav.2020.04.001 33100820 PMC7584376

[6] Kaplan H, Thompson RC, Trumble BC et al. Coronary atherosclerosis in indigenous south American Tsimane: a cross-sectional cohort study. *Lancet.* 2017;389:1730–9. 10.1016/S0140-6736(17)30752-3 28320601 PMC6028773

[7] Jasieńska G, Thune I, Ellison PT. Energetic factors, ovarian steroids and the risk of breast cancer. *Eur J Cancer Prev* 2000;9:231–40. 10.1097/00008469-200008000-00003 10958326

[8] Pontzer H, Wood BM, Raichlen DA. Hunter-gatherers as models in public health: hunter-gatherer health and lifestyle. *Obes Rev* 2018;19:24–35. 10.1111/obr.12785 30511505

[9] Alberts SC, Archie EA, Gesquiere LR et al. The Male-Female Health-Survival Paradox: A Comparative Perspective on Sex Differences in Aging and Mortality. Washington, DC: National Academies Press (US), 2014.

[10] Austad SN, Fischer KE. Sex differences in lifespan. *Cell Metab* 2016;23:1022–33. 10.1016/j.cmet.2016.05.019 27304504 PMC4932837

[11] Crimmins EM, Kim JK, Solé-Auró A. Gender differences in health: results from SHARE, ELSA and HRS. *Eur J Pub Health* 2011;21:81–91. 10.1093/eurpub/ckq022 20237171 PMC3023013

[12] Austad SN . Why women live longer than men: sex differences in longevity. *Gend Med* 2006;3:79–92. 10.1016/S1550-8579(06)80198-1 16860268

[13] Zarulli V, Kashnitsky I, Vaupel JW. Death rates at specific life stages mold the sex gap in life expectancy. *Proc Natl Acad Sci USA* 2021;118. 10.1073/pnas.2010588118 PMC8157960 33972417

[14] James SL, Abate D, Abate KH et al. Global, regional, and national incidence, prevalence, and years lived with disability for 354 diseases and injuries for 195 countries and territories, 1990–2017: a systematic analysis for the global burden of disease study 2017. *Lancet.* 2018;392:1789–858. 10.1016/S0140-6736(18)32279-7 30496104 PMC6227754

[15] Mauvais-Jarvis F, Bairey Merz N, Barnes PJ et al. Sex and gender: modifiers of health, disease, and medicine. *Lancet.* 2020;396:565–82. 10.1016/S0140-6736(20)31561-0 32828189 PMC7440877

[16] Crimmins EM, Shim H, Zhang YS et al. Differences between men and women in mortality and the health dimensions of the morbidity process. *Clin Chem* 2019;65:135–45. 10.1373/clinchem.2018.288332 30478135 PMC6345642

[17] Reynolds AZ, Wander K, Sum CY et al. Matriliny reverses gender disparities in inflammation and hypertension among the Mosuo of China. *Proc Natl Acad Sci USA* 2020;117:30324–7. 10.1073/pnas.2014403117 33199598 PMC7720212

[18] Van Schaik CP, Isler K. Life-history evolution in primates. In: Mitani JC, Call J, Kappeler PM et al. (eds.), *The Evolution of Primate Societies*, pp. 220–44. Chicago, IL: The University of Chicago Press, 2012.

[19] Gilissen EP, Leroy K, Yilmaz Z et al. A neuronal aging pattern unique to humans and common chimpanzees. *Brain Struct Funct* 2016;221:647–64. 10.1007/s00429-014-0931-5 25381006

[20] Emery Thompson M, Fox SA, Berghänel A et al. Wild chimpanzees exhibit humanlike aging of glucocorticoid regulation. *Proc Natl Acad Sci USA* 2020;117:8424–30. 10.1073/pnas.1920593117 32229565 PMC7165472

[21] Rosati AG, Hagberg L, Enigk DK et al. Social selectivity in aging wild chimpanzees. *Science.* 2020;370:473–6. 10.1126/science.aaz9129 33093111 PMC7668794

[22] Gurven MD, Gomes CM. 5. Mortality, senescence, and life span. In: Muller MN, Wrangham RW, Pilbeam DR (eds.), *Chimpanzees and Human Evolution*, pp. 181–216. Cambridge, MA and London, England: Harvard University Press, 2017.

[23] Hanamura SH, Kooriyama TA, Hosaka KA. Diseases and deaths: Variety and impact on social life. In: Nakamura M, Hosaka K, Itoh N et al. (eds.), *Mahale Chimpanzees: 50 Years of Research*, pp. 354–71. Cambridge, UK: Cambridge University Press, 2015.

[24] Williams JM, Lonsdorf EV, Wilson ML et al. Causes of death in the Kasekela chimpanzees of Gombe National Park, Tanzania. *Am J Primatol* 2008;70:766–77. 10.1002/ajp.20573 18506732

[25] Lovell NC . An evolutionary framework for assessing illness and injury in nonhuman primates. *Am J Phys Anthropol* 1991;34:117–55. 10.1002/ajpa.1330340608

[26] Nunn CL, Craft ME, Gillespie TR et al. The sociality– health –fitness nexus: synthesis, conclusions and future directions. *Philos Trans R Soc Lond Ser B Biol Sci* 370:20140115. 10.1098/rstb.2014.0115 PMC4410381 25870401

[27] Peters A, Delhey K, Nakagawa S et al. Immunosenescence in wild animals: Meta-analysis and outlook. *Ecol Lett* 2019;22:1709–22. 10.1111/ele.13343 31321874

[28] Shanley DP, Aw D, Manley NR et al. An evolutionary perspective on the mechanisms of immunosenescence. *Trends Immunol* 2009;30:374–81. 10.1016/j.it.2009.05.001 19541538

[29] Hawkley LC, Cacioppo JT. Aging and loneliness: downhill quickly? *Curr Dir Psychol Sci* 2007;16:187–91. 10.1111/j.1467-8721.2007.00501.x

[30] Seeman TE, Crimmins E. Social environment effects on health and aging: integrating epidemiologic and demographic approaches and perspectives. *Ann N Y Acad Sci* 2001;954:88–117. 10.1111/j.1749-6632.2001.tb02749.x 11797869

[31] Nishimura Y, Oikawa M, Motegi H. What explains the difference in the effect of retirement on health? Evidence from global aging data: evidence from global aging data. *J Econ Surv* 2018;32:792–847. 10.1111/joes.12215

[32] Rolandi E, Rossi M, Colombo M et al. Lifestyle, cognitive, and psychological factors associated with a resilience phenotype in aging: a multidimensional approach on a population-based sample of oldest-old (80+). *J Gerontol B Psychol Sci Soc Sci* 2024;79:gbae132. 10.1093/geronb/gbae132 39096236 PMC11402365

[33] Thompson González N, Machanda Z, Emery TM. Age-related social selectivity: an adaptive lens on a later life social phenotype. *Neurosci Biobehav Rev* 2023;152:105294. 10.1016/j.neubiorev.2023.105294 37380041 PMC10529433

[34] Bronikowski AM, Altmann J, Brockman DK et al. Aging in the natural world: comparative data reveal similar mortality patterns across primates. *Science* 2011;331:1325–8. 10.1126/science.1201571 21393544 PMC3396421

[35] Trivers RL . Parental investment and sexual selection introduction. *Sexual Selection and the Descent of Man* 1972;1871-1971:136–207. 10.4324/9781315129266-7

[36] Kaplan H, Hill K, Lancaster J et al. A theory of human life history evolution: diet, intelligence, and longevity. *Evol Anthropol* 2000;9:156–85. 10.1002/1520-6505(2000)9:4<156::AID-EVAN5>3.0.CO;2-7

[37] Mariencheck CL . The immunity gap in primates. *Evol Anthropol* 2024;33:e22038. 10.1002/evan.22038 38877873

[38] Stearns SC . Trade-offs in life-history evolution. *Funct Ecol* 1989;3:259–68. 10.2307/2389364

[39] Zuk M, Stoehr AM. Immune defense and host life history. *Am Nat* 2002;160:S9–22. 10.1086/342131 18707455

[40] Bateman A, Innes J. Intra-sexual selection in drosophila. *Heredity (Edinb)* 1948;2:349–68. 10.1038/hdy.1948.21 18103134

[41] Rolff J . Bateman’s principle and immunity. *Proc Biol Sci* 2002;269:867–72. 10.1098/rspb.2002.1959 11958720 PMC1690964

[42] Williams PD, Day T. Antagonistic pleiotropy, mortality source interactions, and the evolutionary theory of senescence. *Evolution.* 2003;57:1478–88. 10.1111/j.0014-3820.2003.tb00356.x 12940353

[43] Alberts SC, Altmann J. Matrix models for primate life history analysis. In: Kappeler P, Pereira M (eds.), *Primate Life Histories and Socioecology*, pp. 66–102. Chicago, IL: University of Chicago Press, 2003.

[44] Bronikowski AM, Cords M, Alberts SC et al. Female and male life tables for seven wild primate species Background & Summary. *Scientific Data* 2016;3:160006. 10.1038/sdata.2016.6 26928014 PMC4772651

[45] Gillespie TR, Nunn CL, Leendertz FH. Integrative approaches to the study of primate infectious disease: implications for biodiversity conservation and global health. *Am J Phys Anthropol* 2008;137:53–69. 10.1002/ajpa.20949 19003885

[46] Lukasik M . Establishing a long-term veterinary project for free-ranging chimpanzees in Tanzania. *Pan Afr News* 2002;9:13–7. 10.5134/143417

[47] Travis DA, Lonsdorf EV, Mlengeya T et al. A science-based approach to managing disease risks for ape conservation. *Am J Primatol* 2008;70:745–50. 10.1002/ajp.20566 18506726

[48] Negrey JD, Emery Thompson M, Dunn CD et al. Female reproduction and viral infection in a long-lived mammal. *J Anim Ecol* 2022;91:1999–2009. 10.1111/1365-2656.13799 35988037 PMC9532343

[49] Henning K . What is syndromic surveillance? *MMWR Suppl* 2004;53:5–11. 15714620

[50] Stedman’s. Stedman’s Medical Dictionary for the Health Professions and Nursing5th edn. Philadelphia, PA: Lippincott Williams and Wilkins, 2004. 2154.

[51] Wood BM, Watts DP, Mitani JC et al. Favorable ecological circumstances promote life expectancy in chimpanzees similar to that of human hunter-gatherers. *J Hum Evol* 2017;105:41–56. 10.1016/j.jhevol.2017.01.003 28366199 PMC5526083

[52] Muller MN, Sabbi KH, Thompson ME et al. Age-related reproductive effort in male chimpanzees: terminal investment or alternative tactics? *Anim Behav* 2024;213:11–21. 10.1016/j.anbehav.2024.04.002 39007109 PMC11238624

[53] Emery, Thompson M, Sabbi K. Evolutionary demography of the great apes. In Burger O, Lee R, Sear R (eds.), *Human Evolutionary Demography*, pp. 423–74. Cambridge, UK: Open Book Publishers, 2024.

[54] Van Lawick-goodall J . The behaviour of free-living chimpanzees in the Gombe stream reserve. *Anim Behav Monogr* 1968;1:161–311–IN12. 10.1016/S0066-1856(68)80003-2

[55] Lonsdorf EV, Stanton MA, Pusey AE et al. Sources of variation in weaned age among wild chimpanzees in Gombe National Park, Tanzania. *Am J Phys Anthropol [Internet]* 2020;171:419–29. 10.1002/ajpa.23986 31845329

[56] Pusey AE . Behavioral changes at adolescence in chimpanzees. *Behaviour.* 1990;115:203–46. 10.1163/156853990X00581

[57] Muller MN, Blurton Jones NG, Colchero F et al. Sexual dimorphism in chimpanzee (pan troglodytes schweinfurthii) and human age-specific fertility. *J Hum Evol* 2020;144:102795. 10.1016/j.jhevol.2020.102795 32454364 PMC7337577

[58] Thompson ME, Kahlenberg SM, Gilby IC et al. Core area quality is associated with variance in reproductive success among female chimpanzees at Kibale National Park. *Anim Behav* 2007;73:501–12. 10.1016/j.anbehav.2006.09.007

[59] Murray CM, Mane SV, Pusey AE. Dominance rank influences female space use in wild chimpanzees, pan troglodytes: towards an ideal despotic distribution. *Anim Behav* 2007;74:1795–804. 10.1016/j.anbehav.2007.03.024

[60] Wrangham RW, Smuts BB. Sex differences in the behavioural ecology of chimpanzees in the Gombe National Park, Tanzania. *J Reprod Fertil Suppl* 1980;Suppl 28:13–31. 6934308

[61] Muller MN . Agonistic relations among Kanyawara chimpanzees. In: Boesch C, Hohmann G (eds.), *Behavioural Diversity in Chimpanzees and Bonobos*, pp. 112–24. Cambridge: Cambridge University Press, 2002.

[62] Muller MN, Kahlenberg SM, Emery Thompson M et al. Male coercion and the costs of promiscuous mating for female chimpanzees. *Proc Biol Sci* 2007;274:1009–14. 10.1098/rspb.2006.0206 17264062 PMC2141672

[63] Wilson ML, Boesch C, Fruth B et al. Lethal aggression in pan is better explained by adaptive strategies than human impacts. *Nature.* 2014;513:414–7. 10.1038/nature13727 25230664

[64] Pusey AE, Schroepfer-Walker K, Trivers R et al. Female competition in chimpanzees. *Philos Trans R Soc Lond Ser B Biol Sci* 2013;368:20130077. 10.1098/rstb.2013.0077 24167307 PMC3826206

[65] Walker KK, Foerster S, Murray CM et al. Evaluating adaptive hypotheses for female-led infanticide in wild chimpanzees. *Anim Behav* 2021;180:23–36. 10.1016/j.anbehav.2021.07.025

[66] Lonsdorf EV, Gillespie TR, Wolf TM et al. Socioecological correlates of clinical signs in two communities of wild chimpanzees (pan troglodytes) at Gombe National Park, Tanzania. *Am J Primatol* 2018;80:e22562. 10.1002/ajp.22562 PMC5112147 27182786

[67] Chapman CA, Wrangham RW, Emery Thompson M et al. Risk factors for respiratory illness in a community of wild chimpanzees ( pan troglodytes schweinfurthii ). *R Soc Open Sci* 2018;5. 10.1098/rsos.180840 PMC6170528 30839693

[68] Köndgen S, Kühl H, N’Goran PK et al. Pandemic human viruses cause decline of endangered great apes. *Curr Biol* 2008;18:260–4. 10.1016/j.cub.2008.01.012 18222690

[69] Lonsdorf EV, Murray CM, Lonsdorf EV et al. A retrospective analysis of factors correlated to chimpanzee (pan troglodytes schweinfurthii) respiratory health at Gombe National Park, Tanzania. *Ecohealth* 2011;8:26–35. 10.1007/s10393-011-0683-0 21562902

[70] Negrey JD, Reddy RB, Scully EJ et al. Simultaneous outbreaks of respiratory disease in wild chimpanzees caused by distinct viruses of human origin. *Emerg Microbes Infect* 2019;8:139–149. 10.1080/22221751.2018.1563456 PMC6455141 30866768

[71] Scully EJ, Basnet S, Wrangham RW et al. Lethal respiratory disease associated with human rhinovirus C in wild chimpanzees, Uganda, 2013. *Emerg Infect Dis* 2018;24:267–74. 10.3201/eid2402.170778 29350142 PMC5782908

[72] Chi F, Leider M, Leendertz F et al. New *Streptococcus pneumoniae* clones in deceased wild chimpanzees. *J Bacteriol* 2007;189:6085–8. 10.1128/JB.00468-07 17586649 PMC1952052

[73] Köndgen S, Leider M, Lankester F et al. Pasteurella multocida involved in respiratory disease of wild chimpanzees. *PLoS One* 2011;6:e24236. 10.1371/journal.pone.0024236 21931664 PMC3169569

[74] Kirchhoff CA . Life and Death in the Gombe Chimpanzees2019th edn. Cham, Switzerland: Springer Nature, 2019. 181 (Developments in Primatology: Progress and Prospects).

[75] Parsons MB, Travis D, Lonsdorf EV et al. Epidemiology and molecular characterization of cryptosporidium spp. in humans, wild primates, and domesticated animals in the greater Gombe ecosystem, Tanzania. *PLoS Negl Trop Dis* 2015;9. 10.1371/journal.pntd.0003529 PMC4336292 25700265

[76] Deere JR, Parsons MB, Lonsdorf EV et al. Entamoeba histolytica infection in humans, chimpanzees and baboons in the greater Gombe ecosystem, Tanzania. *Parasitology* 2019;146:1116–22. 10.1017/S0031182018001397 30157971 PMC12938633

[77] Gillespie TR, Lonsdorf EV, Canfield EP et al. Demographic and ecological effects on patterns of parasitism in eastern chimpanzees (pan troglodytes schweinfurthii) in Gombe National Park, Tanzania. *Am J Phys Anthropol* 2010;143:534–44. 10.1002/ajpa.21348 20623606 PMC4048996

[78] Terio KA, Lonsdorf EV, Kinsel MJ et al. Oesophagostomiasis in non-human primates of Gombe National Park, Tanzania. *Am J Primatol* 2018;80:e22572. 10.1002/ajp.22572 PMC5161720 27309976

[79] Strahan EK, Witherbee J, Bergl R et al. Potentially zoonotic enteric infections in gorillas and chimpanzees, Cameroon and Tanzania. *Emerg Infect Dis* 2024;30:577–80. 10.3201/eid3003.230318 38407249 PMC10902540

[80] Pusey AE, Wilson ML, Anthony CD. Human impacts, disease risk, and population dynamics in the chimpanzees of Gombe National Park, Tanzania. *Am J Primatol* 2008;70:738–44. 10.1002/ajp.20567 18521891

[81] Lonsdorf EV, Anderson KE, Stanton MA et al. Boys will be boys: sex differences in wild infant chimpanzee social interactions. *Anim Behav* 2014;88:79–83. 10.1016/j.anbehav.2013.11.015 24489384 PMC3904494

[82] Lonsdorf EV, Travis D, Pusey AE et al. Using retrospective health data from the Gombe chimpanzee study to inform future monitoring efforts. *Am J Primatol* 2006;68:897–908. 10.1002/ajp.20296 16900499

[83] Wrangham RW, Chapman CA, Clark-Arcadi AP et al. Social ecology of Kanyawara chimpanzees: Implications for understanding the costs of great ape groups. In: McGrew WC, Marchant LF, Nishida T (eds.), *Great Ape Societies*, pp. 45–57. Cambridge: Cambridge University Press, 1996.

[84] Strier KB . Long-term field studies: positive impacts and unintended consequences. *Am J Primatol* 2010;72:772–8. 10.1002/ajp.20830 20653002

[85] Goodall J . The Chimpanzees of Gombe: Patterns of Behavior. Cambridge, MA: Harvard University Press, 1986.

[86] Nishida T . The social group of wild chimpanzees in the Mahali Mountains. *Primates.* 1968;9:167–224. 10.1007/BF01730971

[87] R Foundation for Statistical Computing . R: A Language and Environment for Statistical Computing [Internet]. Vienna, Austria: R Core Team, 2020. Available from: https://www.R-project.org/.

[88] Posit Team R. RStudio: Integrated development environment for R. Posit Software, PBC, Boston, MA. 2024. http://www.posit.co/

[89] Brooks ME, Kristensen K, van Benthem KJ et al. Modeling zero-inflated count data with glmmTMB BioRxiv 2017:132753. 10.1101/132753

[90] Hartig F . DHARMa: Residual Diagnostics for Hierarchical (Multi-Level / Mixed) Regression Models 2020. R package version 0.4. 6 [Internet].

[91] Fox J, Weisberg S. An R Companion to Applied Regression3rd edn. Christchurch, New Zealand: Sage Publications, 2018. 608.

[92] Lüdecke D, Ben-Shachar M, Patil I et al. Performance: an R package for assessment, comparison and testing of statistical models. *J Open Source Softw* 2021;6:3139. 10.21105/joss.03139

[93] Shumway R, Stoffer D. Time Series: A Data Analysis Approach Using R [Internet]. London, England: CRC Press, 2019. 272 Available from: 10.1201/9780429273285/time-series-robert-shumway-david-stoffer.

[94] Feldblum JT, Manfredi S, Gilby IC et al. The timing and causes of a unique chimpanzee community fission preceding Gombe’s “four-year war”. *Am J Phys Anthropol* 2018;166:730–744. 10.1002/ajpa.23462 29566432

[95] Brooks ME, Kristensen K, van Benthem KJ et al. glmmTMB balances speed and flexibility among packages for zero-inflated generalized linear mixed modeling. *The R journal* 2017;9:378–400. 10.32614/RJ-2017-066

[96] Kaur T, Singh J, Tong S et al. Descriptive epidemiology of fatal respiratory outbreaks and detection of a human-related metapneumovirus in wild chimpanzees (pan troglodytes) at Mahale Mountains National Park, western Tanzania. *Am J Primatol* 2008;70:755–65. 10.1002/ajp.20565 18548512 PMC7159556

[97] Ursin RL, Klein SL. Sex differences in respiratory viral pathogenesis and treatments. *Annu Rev Virol* 2021;8:393–414. 10.1146/annurev-virology-091919-092720 34081540

[98] Arlet ME, Carey JR, Molleman F. Species, age and sex differences in type and frequencies of injuries and impairments among four arboreal primate species in Kibale National Park, Uganda. *Primates.* 2009;50:65–73. 10.1007/s10329-008-0119-9 19067112

[99] MacCormick HA, MacNulty DR, Bosacker AL et al. Male and female aggression: lessons from sex, rank, age, and injury in olive baboons. *Behav Ecol* 2012;23:684–691. 10.1093/beheco/ars021

[100] Massaro AP, Lonsdorf EV, Mwacha D et al. Genital wounding in chimpanzees (pan troglodytes): targeted attacks or happenstance? *Int J Primatol* 2024;46:145–57. 10.1007/s10764-024-00454-2 PMC13035354 41919273

[101] Stanford C, Wallis J, Mpongo E et al. Hunting decisions in wild chimpanzees. *Behaviour.* 1994;131:1–18. 10.1163/156853994X00181

[102] Uehara S . Predation on mammals by the chimpanzee (pan troglodytes). *Primates.* 1997;38:193–214. 10.1007/BF02382009

[103] Watts D, Mitani J. Boundary patrols and intergroup encounters in wild chimpanzees. *Behaviour.* 2001;138:299–327. 10.1163/15685390152032488

[104] Jurmain R . Skeletal evidence of trauma in African apes, with special reference to the Gombe chimpanzees. *Primates.* 1997;38:1–14. 10.1007/BF02385918

[105] Novak SA, Hatch MA. 13 intimate wounds: Craniofacial trauma inWomen and female chimpanzees. In: Muller M, Wrangham RW (eds.), *Sexual Coercion in Primates and Humans*, pp. 322–45. Cambridge, MA and London, England: Harvard University Press, 2009.

[106] Cords M, Arguelles N. Costs of social conflict: do injuries mirror patterns of aggression in blue monkeys? *Behav Ecol Sociobiol* 2023;77:106. 10.1007/s00265-023-03382-y

[107] Feder JA, Lu A, Koenig A et al. The costs of competition: injury patterns in 2 Asian colobine monkeys. *Behav Ecol* 2019;30:1242–1253. https://academic.oup.com/beheco/article-abstract/30/5/1242/5492507

[108] Kumar V, Sankhyan V. Musculoskeletal injuries: prevalence and severity in free-range rhesus macaques (Macaca mulatta) of Himalayan Shivalik Hills, northern India. *Biol Rhythm Res* 2021;52:1044–54. 10.1080/09291016.2019.1613795

[109] Archer J . Sex differences in aggression in real-world settings: a meta-analytic review. *Rev Gen Psychol* 2004;8:291–322. 10.1037/1089-2680.8.4.291

[110] Sorenson SB . Gender disparities in injury mortality: consistent, persistent, and larger than you’d think. *Am J Public Health* 2011;101:S353–8. 10.2105/AJPH.2010.300029 21778511 PMC3222499

[111] Foerster S, Franz M, Murray CM et al. Chimpanzee females queue but males compete for social status. *Sci Rep* 2016;6:35404. 10.1038/srep35404 27739527 PMC5064376

[112] Thompson González N, Machanda Z, Otali E et al. Age-related change in adult chimpanzee social network integration. *Evol Med Public Health* 2021;9:448–59. 10.1093/emph/eoab040 34987824 PMC8697844

[113] Massaro AP, Wroblewski EE, Mjungu DC et al. Monopolizability of mating opportunities promotes within-community killing in chimpanzees. *SSRN Electron J* 2021. 10.2139/ssrn.3974936

[114] Negrey JD, Thompson ME, Langergraber KE et al. Demography, life-history trade-offs, and the gastrointestinal virome of wild chimpanzees. *Philos Trans R Soc Lond Ser B Biol Sci* 2020;375:20190613. 10.1098/rstb.2019.0613 32951554 PMC7540950

[115] Munir G, Nealen P. Survey of captive hylobatid diets and their association with gastrointestinal distress. *Journal of Zoo and Aquarium Research* 2021;9:73–80.

[116] Gottlieb DH, Del Rosso L, Sheikhi F et al. Personality, environmental stressors, and diarrhea in rhesus macaques: an interactionist perspective. *Am J Primatol* 2018;80:e22908. 10.1002/ajp.22908 30152539 PMC6705421

[117] Jia L, Weng S, Wu J et al. Preexisting antibodies targeting SARS-CoV-2 S2 cross-react with commensal gut bacteria and impact COVID-19 vaccine induced immunity. *Gut Microbes* 2022;14:2117503. 10.1080/19490976.2022.2117503 36100957 PMC9481142

[118] Melchior K, Gerner RR, Hossain S et al. IL-22-dependent responses and their role during Citrobacter rodentium infection. *Infect Immun* 2024;92:e0009924. 10.1128/iai.00099-24 38557196 PMC11075456

[119] Hou G, Son J, Gomez Castro MF et al. Innate immune sensing of rotavirus by intestinal epithelial cells leads to diarrhea. *Cell Host Microbe* 2025;33:408–419.e8. 10.1016/j.chom.2025.02.005 40037352 PMC11932023

[120] Phillips SR, Goldberg TL, Muller MN et al. Faecal parasites increase with age but not reproductive effort in wild female chimpanzees. *Philos Trans R Soc Lond Ser B Biol Sci* 2020;375:20190614. 10.1098/rstb.2019.0614 32951547 PMC7540947

[121] Gilby IC, Machanda ZP, O’Malley RC et al. Predation by female chimpanzees: toward an understanding of sex differences in meat acquisition in the last common ancestor of pan and homo. *J Hum Evol* 2017;110:82–94. 10.1016/j.jhevol.2017.06.015 28778463 PMC5570454

[122] Gilby IC, Machanda ZP, Mjungu DC et al. “Impact hunters” catalyse cooperative hunting in two wild chimpanzee communities. *Philos Trans R Soc Lond Ser B Biol Sci* 2015;370:20150005. 10.1098/rstb.2015.0005 26503679 PMC4633842

[123] Muehlenbein MP, Watts DP. The costs of dominance: testosterone, cortisol and intestinal parasites in wild male chimpanzees. *Biopsychosoc Med* 2010;4:21. 10.1186/1751-0759-4-21 21143892 PMC3004803

[124] Muller MN . Testosterone and reproductive effort in male primates. *Horm Behav* 2017;91:36–51. 10.1016/j.yhbeh.2016.09.001 27616559 PMC5342957

[125] Deere JR, Lonsdorf EV, Clennon JA et al. Bridging the gap: integrating knowledge from the study of social network analysis and infectious disease dynamics in human and nonhuman primates. *Annu Rev Anthropol* 2024;53:37–53. 10.1146/annurev-anthro-052721-085447 41048884 PMC12494230

[126] GBD 2016 Diarrhoeal Disease Collaborators . Estimates of the global, regional, and national morbidity, mortality, and aetiologies of diarrhoea in 195 countries: a systematic analysis for the global burden of disease study 2016. *Lancet Infect Dis* 2018;18:1211–28. 30243583 10.1016/S1473-3099(18)30362-1 PMC6202444

[127] Kyu HH, Vongpradith A, Dominguez RMV et al. Global, regional, and national age-sex-specific burden of diarrhoeal diseases, their risk factors, and aetiologies, 1990–2021, for 204 countries and territories: a systematic analysis for the global burden of disease study 2021. *Lancet Infect Dis* 2025;25:519–536. Available from: http://www.thelancet.com/article/S1473309924006911/abstract 10.1016/S1473-3099(24)00691-1 PMC12018300 39708822

[128] Wrangham RW . Feeding behaviour of chimpanzees in Gombe National Park, Tanzania. In: Clutton-Brock TH (ed.), *Primate Ecology: Studies of Feeding and Ranging Behavior in Lemurs, Monkey and Apes*, pp. 503–38. London & New York: Elsevier, 1977.

[129] Whitacre C . Sex differences in autoimmune disease. *Nat Immunol* 2001;2:777–80. 10.1038/ni0901-777 11526384

[130] Alzheimer . 2025 Alzheimer’s disease facts and figures. *Alzheimer’s Assocation* 2025;21:31–7.

[131] Fillingim RB, King CD, Ribeiro-Dasilva MC et al. Sex, gender, and pain: a review of recent clinical and experimental findings. *J Pain* 2009;10:447–85. 10.1016/j.jpain.2008.12.001 19411059 PMC2677686

[132] Keestra SM, Male V, Salali GD. Out of balance: the role of evolutionary mismatches in the sex disparity in autoimmune disease. *Med Hypotheses* 2021;151:110558. 10.1016/j.mehy.2021.110558 33964604

[133] Dias SP, Brouwer MC, van de Beek D. Sex and gender differences in bacterial infections. *Infect Immun* 2022;90:e0028322. 10.1128/iai.00283-22 36121220 PMC9584217

[134] Lea AJ, Clark AG, Dahl AW et al. Applying an evolutionary mismatch framework to understand disease susceptibility. *PLoS Biol* 2023;21:e3002311. 10.1371/journal.pbio.3002311 37695771 PMC10513379

[135] Emery Thompson M . Evolutionary approaches in aging research. *Cold Spring Harb Perspect Med* 2022;12:a041195. Available from: https://perspectivesinmedicine.cshlp.org/content/12/11/a041195.short 10.1101/cshperspect.a041195 PMC9619358 36041879

[136] Bolton JL . Menopausal hormone therapy, age, and chronic diseases: perspectives on statistical trends. *Chem Res Toxicol* 2016;29:1583–90. 10.1021/acs.chemrestox.6b00272 27636306 PMC5069683

[137] Wood BM, Negrey JD, Brown JL et al. Demographic and hormonal evidence for menopause in wild chimpanzees. *Science.* 2023;382:eadd5473. 10.1126/science.add5473 37883540 PMC10645439

[138] Groeneveld JM, Ballering AV, van Boven K et al. Sex differences in incidence of respiratory symptoms and management by general practitioners. *Fam Pract* 2020;37:631–6. 10.1093/fampra/cmaa040 32473018 PMC7571773

[139] Allen DJ, Sesti F. Health inequalities and women. *British Medical Association* 2018;56:10.

[140] Vaupel JW, Yashin AI. Heterogeneity’s ruses: some surprising effects of selection on population dynamics. *Am Stat* 1985;39:176–85. 12267300

